# Repurposing Pitavastatin and L-Glutamine: Replenishing β-Cells in Hyperlipidemic Type 2 Diabetes Mouse Model

**DOI:** 10.3390/life13040929

**Published:** 2023-04-01

**Authors:** Sayantani Pramanik Palit, Roma Patel, Nishant Parmar, Nirali Rathwa, Nilay Dalvi, A. V. Ramachandran, Rasheedunnisa Begum

**Affiliations:** 1 Department of Biochemistry, Faculty of Science, The Maharaja Sayajirao University of Baroda, Vadodara 390002, Gujarat, India; 2 School of Liberal Studies and Education, Navrachana University, Vadodara 391410, Gujarat, India

**Keywords:** obesity, β-cell regeneration, mitochondrial biogenesis, adiponectin, GLP-1

## Abstract

Type 2 diabetes (T2D) is associated with obesity and declining β-cells. L-glutamine has been implicated in the amelioration of T2D by virtue of its incretin secretagogue property while, there are mixed reports on pitavastatin’s adiponectin potentiating ability. We aimed to investigate the effect of pitavastatin (P), L-glutamine (LG), and combination (P + LG) on glycemic control and β-cell regeneration in a high-fat diet (HFD) + streptozotocin (STZ)-induced T2D mouse model. C57BL6/J mice treated with HFD + STZ were divided into four groups: diabetes control (HFD + STZ), P, LG, and P + LG, while the control group (NCD) was fed with the normal-chow diet. Significant amelioration was observed in the combination therapy as compared to monotherapies in respect of (i) insulin resistance, glucose intolerance, lipid profile, adiponectin levels, and mitochondrial complexes I, II, and III activities, (ii) reduced phosphoenolpyruvate carboxykinase, glucose 6-phophatase, glycogen phosphorylase, and GLUT2 transcript levels with increased glycogen content in the liver, (iii) restoration of insulin receptor 1β, pAkt/Akt, and AdipoR1 protein levels in skeletal muscle, and (iv) significant increase in islet number due to β-cell regeneration and reduced β-cell death. L-glutamine and pitavastatin in combination can ameliorate T2D by inducing β-cell regeneration and regulating glucose homeostasis.

## 1. Introduction

Insulin resistance, reduced insulin secretion, dyslipidemia, and β-cell degeneration lay the foundation of type 2 diabetes (T2D). Against a backdrop of obesity or genetic factors the risk for these hallmark foundations increases many folds. While there is little that can be done to reduce the genetic predisposition, having a healthy and active lifestyle can definitely reduce the associated risks. T2D takes several years to set in with the pre-diabetic stage typically being long; however, this stage being asymptomatic, most cases are detected only after diabetes is established [1]. In the prediabetes stage, as insulin resistance sets in and the first phase of insulin response starts to diminish, resulting in β-cells overworking to compensate for the reduced insulin levels. In addition, the excessive circulating lipids in obese conditions create a stressful microenvironment resulting in eventual loss of β-cell mass. Studies have shown that by the time T2D is diagnosed, islet function is already reduced by 50% and β-cell mass by 60% due to the increased stress and accelerated apoptosis [2,3]. Further, in cadaver pancreatic autopsy of obese patients with prediabetes and T2D, Butler et al. demonstrated a 40% reduction in β-cell mass, as compared to obese individuals without T2D [4]. Impaired action or a 50% drop in incretin hormones, glucose-dependent insulinotropic polypeptide (GIP), and GLP-1 have also been reported to result in the decline of β-cell function in type 2 diabetes. This potentiates close to 70% of the meal-induced insulin response in healthy individuals. In this context several secretagogues or mimetics have been studied and are in practice as T2D therapeutic modalities [1,4]. In addition, lipid-lowering drugs or drugs that delay gastric emptying are also prescribed as a parallel solution. While newer molecules are constantly being researched to find better solutions to this multifactorial disease, we believe that the presently available on-shelf drugs or nutraceutics have an untapped potential to reverse and restore glucose homeostasis. In this context, though, L-glutamine is not a prescribed drug for T2D but is consumed by athletes as a supplement to enhance or maintain their power output and strength, and as a matter of fact, L-glutamine is also a GLP-1 secretagogue that works by raising cytosolic Ca^2+^ and cAMP in intestinal L-cells [2,5]. Interestingly, L-glutamine levels in prediabetics were reported to be higher, while they were found to be low in T2D subjects. The fasting plasma levels of L-glutamine were found to have a significant association with both insulin secretion and sensitivity [6,7]. Another class of drugs, statins is used for the comprehensive management of hypercholesterolemia-associated obesity-induced T2D. Though statins are debated to have diabetogenic properties, these clinical studies or meta-analyses clearly suggest the statin-induced diabetes to be due to a relatively high dose of statin which is generally recommended to dyslipidemic or obese individuals. Moreover, out of the seven types of statins, pitavastatin has shown a remarkable outcome on the glycemic control in T2D patients compared to other members of the statin group [8,9].

Thus, we hypothesized that the combination of two small molecules, i.e., pitavastatin and L-glutamine can ameliorate high-fat diet (HFD)- and streptozotocin (STZ)-induced T2D in C57BL/6J mice.

## 2. Materials and Methods

### 2.1. Animals and Experimental Strategy

Nine- to ten-week-old (22 g) male C57BL/6 mice procured from the Advanced Centre for Treatment, Research and Education in Cancer (Navi-Mumbai, India) were housed in our animal house (Department of Biochemistry, The Maharaja Sayajirao University of Baroda, Vadodara, Gujarat, India), and maintained in a 12 h:12 h light–dark cycle, with free access to normal-chow diet (NCD)/high-fat diet (HFD) (Keval Sales Corporation, India) and water ad libitum. 

**Figure A1:**
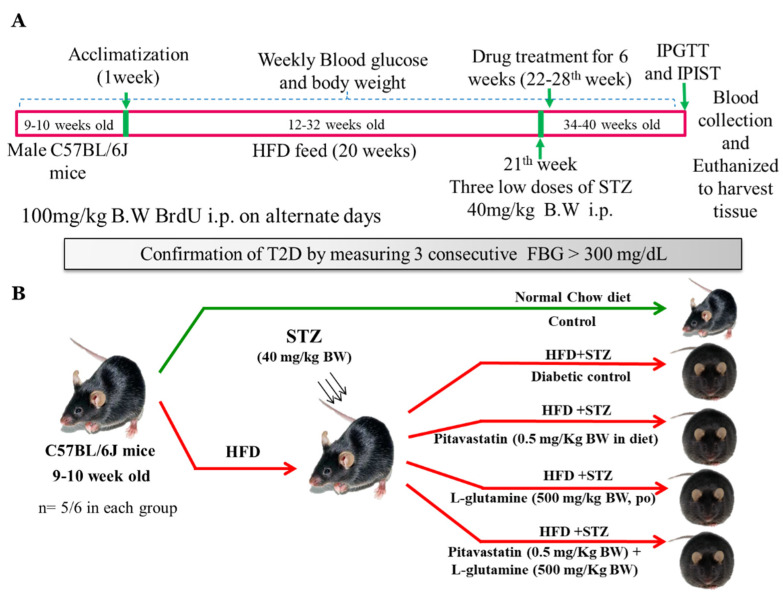
(**A**) The experimental timeline: ~9–10 weeks old male C57BL/6J mice were subjected to 1 week acclimatization and weekly food intake, water consumption, and BW and FBG levels were monitored throughout the study period. Post-acclimatization they were divided into two groups: control group was fed with normal-chow diet (NCD) and 20 weeks of HFD treatment was given to the second group to induce obesity and insulin-resistance. On the 21st week, the HFD-fed mice received three consecutive doses of STZ (40 mg/kg i.p.) to induce β-cell loss. T2D was confirmed with FBG measuring > 240 mg/dL. The T2D animals were further randomly divided into four groups for 6 weeks therapy. BrdU (100 mg/Kg b.w. i.p.) was administered on alternate days for the entire treatment period. The animals were then euthanized and tissues were harvested for further analysis. (**B**) Strategy for drug treatment: Animals were divided into two groups; one control group, fed with normal-chow diet (NCD, n = 6) and another T2D group (HFD + STZ, n = 25) which was further divided into four groups (n = 6/group): HFD + STZ (diabetic control), pitavastatin (0.5 mg/kg b.w. in diet), L-glutamine (500 mg/kg b.w. p.o.), and pitavastatin (0.5 mg/kg b.w. in diet) + L-glutamine (500 mg/kg b.w. p.o.). The drugs were administered daily for 6 weeks.

Based on the blood glucose levels and body weight, the animals were first divided randomly into two groups: one fed a normal-chow diet (NCD) (n = 6) and the other a high-fat diet (HFD) (n = 25) to develop obesity-induced insulin resistance. Nutritional composition shown in Table A4. After 20 weeks the HFD mice were administered with three consecutive doses of streptozotocin (STZ) (MP Biomedicals, India) (40 mg/kg i.p.) to induce β-cell loss [10]. HFD + STZ-induced T2D was confirmed by measuring the body weight (b.w.) (≥30 g) and three consecutive readings of fasting blood glucose (FBG) (≥240 mg/dL) (Figure A1A) on the 146th, 150^th^, and 153rd days. 

The mice had turned diabetic by the end of 21st week and on 22nd week, the HFD + STZ group was further randomly divided into four groups (n = 6/group): (i) HFD + STZ (diabetic control), (ii) pitavastatin (P, 0.5 mg/kg b.w. in diet) [11], (iii) L-glutamine (LG, 500 mg/kg b.w. p.o.) [12,13], and (iv) pitavastatin (0.5 mg/kg b.w. in diet) + L-glutamine (P + LG, 500 mg/kg b.w. p.o.) treated (Figure A1B). The treatment was continued daily for six weeks along with 5-bromo-2’-deoxyuridine (BrdU) (MP Biomedicals, Mumbai, India) on alternate days (100 mg/kg b.w. i.p.). FBG and BW of the animals were measured weekly using a glucometer (TRUEresult^®^ Nipro, Osaka, Japan) and a weighing scale, respectively. Simultaneously, food and water intake were also measured. After six weeks of drug treatment, glucose tolerance and insulin tolerance were evaluated by an intraperitoneal glucose-tolerance test (IGTT) and an intraperitoneal insulin-sensitivity test (IPIST). For these, mice were fasted for six hours and then injected with glucose (2 g/kg b.w.) for IPGTT and insulin (0.5 U/kg) for IPIST [14,15]. On the 29th week, 800–900 µL blood was collected from orbital sinus and animals were euthanized for tissue collection by cervical dislocation after subjecting to a mild chloroform chamber. Plasma was separated and stored for further biochemical analysis (Figure A1A). The results for FBG and BW were compared between pre-treatment, i.e., at 21st week before administration of the drugs and post-treatment, i.e., in the 29th week on completing treatment.

All the procedures followed the institutional guidelines approved by Institutional Ethical Committee for Animal Research (IECHR), Faculty of Science, The Maharaja Sayajirao University of Baroda, Vadodara, Gujarat, India (MSU/BIOCHEMISTRY/IAEC/2018-21).

### 2.2. Assessment of Metabolic and Biochemical Parameters

#### 2.2.1. Lipid Profiling

Plasma was used for lipid profiling (total cholesterol, triglycerides, and high-density lipoprotein) using commercially available kits (Reckon Diagnostics Pvt. Ltd., Vadodara, India). Friedewald’s (1972) formula was used for calculating low-density-lipoprotein (LDL) levels [16]. The results are expressed as mg/mL.

#### 2.2.2. Assessment of Plasma Insulin and Adiponectin Levels

Plasma insulin and adiponectin levels were determined by mouse ELISA kits (RayBioiotech, Peachtree Corners, GA, USA) as per manufacturer’s instructions. The results are expressed as mg/mL.

### 2.3. Assessment of Transcript Levels

Liver and skeletal muscle were collected and stored in RNAlater™ Stabilization Solution (Thermo Fisher Scientific, Waltham, MA, USA) and total RNA was extracted using the Trizol method, as described previously. The transcript levels of target genes and GAPDH were monitored by LightCycler^®^480 Real-time PCR (Roche Diagnostics GmbH, Mannheim, Germany) using gene-specific primers (Eurofins, Bangaluru, India) as shown in Table A1. Results are reported as a relative expression normalized with the GAPDH housekeeping gene. The fold variation was determined using the 2^−ΔΔCt^ method according to previously published protocol [17].

### 2.4. Glucoregulatory Enzymes Activities and Liver Glycogen Content

Liver was harvested, snap-frozen using dry ice and stored at −80 °C. Then, 50 mg of liver tissue was homogenized and tissue lysates were used for assaying enzyme activities and determining the glycogen content. Enzyme assays for glucokinase (GCK), fructose 1,6-bisphosphatase (FBPase), phosphoenolpyruvate carboxykinase (PEPCK), and glycogen content were carried out using commercially available kits (BioVision, Milpitas, CA, USA) according to the manufacturer’s protocol. The results are expressed as mg/mL.

### 2.5. Mitochondria Isolation from Skeletal Muscle and Estimation of Oxygen Consumption Rate (OCR)

For OCR studies, skeletal muscle was harvested post-euthanizing and stored in mitochondrial respiration buffer for mitochondria isolation. Mitochondria were isolated from 120 mg skeletal muscle using a mitochondria isolation kit (Thermo Scientific^TM^, Catalog no. 89801) as per manufacturer’s protocol. The isolated mitochondria were resuspended in mitochondria respiration buffer (110 mM Sucrose, 0.5 mM EGTA, 70 mM KCl, 0.1% FFA free BSA, 20 mM HEPES, 3 mM MgCl_2_, and 10 mM KH_2_PO_4_, 20 mM Taurine). The outer membrane integrity of the isolated mitochondria was assessed by impermeability to exogenous cytochrome c and was consistently greater than 95%. Activities of respiratory chain complexes I–III were recorded using 100 µL of substrates (100 mM pyruvate and 800 mM malate (complex I), 1 M succinate (complex II), and 10 mM α-glycerophosphate (complex III)) added to 90 µL of mitochondria suspension in respiration buffer. Other respiration reagents used were 100 mM adenosine diphosphate (100 µL), 1 mM oligomycin (2 µL), 1 mM rotenone (1 µL), and 1 mM antimycin (1 µL). All chemicals were purchased from Sigma-Aldrich, St. Louis, MO, USA. Protein concentration was estimated by the Bradford method [18]. OCR was determined by measuring the amount of oxygen (nmol) consumed, divided by the time elapsed (min) and the amount of protein present in the assay using a Clark oxygen electrode (Hansatech Instruments Ltd., London, UK) [19]. The results are expressed as mg/mL.

### 2.6. Pancreatic Tissue Preparation, Immunohistochemistry (IHC), and Assessment of β-Cell Regeneration and Apoptosis

The pancreas was harvested and fixed in 10% neutral buffered formalin (NBF) for paraffin embedding, and the paraffin-embedded blocks were cut into 5µm sections. Immunofluorescence staining was carried out to detect β-cell regeneration by proliferation (insulin/glucagon/BrdU), neogenesis (insulin/NGN3/PDX1), and α- to β-cell transdifferentiation (insulin/ARX/PAX4). β-cell apoptosis was detected by terminal deoxynucleotidyl transferase dUTP nick end labelling (TUNEL) assay (insulin/fluorochrome-tagged deoxynucleotides) (Thermo Fisher Scientific, MA, USA) and apoptosis-inducing factor (AIF) translocation into the nucleus (insulin/AIF). The sections were deparaffinized in xylene and rehydrated in a series of diluted ethanol (100%, 95%, 80%, and 70%). Slides were then treated with 1 N HCl for 40 min at 37 °C for antigen retrieval followed by blocking in 5% donkey serum diluted in PBST (PBS + 0.1% tween 20) for 1 h at room temperature. The slides were then incubated with the respective primary antibodies prepared in blocking buffer at 37 °C in a humidified chamber for 2 h (guinea pig anti-insulin, rabbit anti-glucagon, rat anti-BrdU, rabbit anti-NGN3, goat anti-PDX1, rabbit anti-ARX, goat anti-PAX4, and rabbit anti-AIF). Sections were washed with PBS and incubated with secondary antibodies prepared in blocking buffer at room temperature for 45 min in dark (donkey anti-guinea pig Alexa Fluor 488, donkey anti-rabbit Alexa Fluor 647, donkey anti-goat Rhodamine Red X, donkey anti-rat Rhodamine Red X, and donkey anti-guinea pig Alexa Fluor 568 (Table A2)). Pancreatic sections were washed with PBS and distilled water, and were mounted with SlowfadeTM Gold Antifade mountant with DAPI (Thermo Fisher Scientific, USA). Coverslips were sealed with nail varnish and stained sections were observed under confocal laser scanning microscope (Olympus FV10i, Tokyo, Japan). Image analysis was carried out in Image J software. Observations were made from three pancreatic sections per group from five different areas. The results are expressed as the percentage positive for fluorescence per field.

### 2.7. Western Blot Analysis

Skeletal muscle was harvested and frozen in dry ice and stored at −80 °C for Western blot analysis of key insulin signaling pathway proteins. The tissue was homogenized in liquid nitrogen and Laemmli buffer (1 M Tris HCl, 10% SDS, 20% glycerol, and 10% β-mercaptoethanol) containing 1 M urea (1:1), protease inhibitor cocktail, and phosphatase inhibitor cocktail 2 and 3 (Sigma, St. Louis, MO, USA). The homogenate was further sonicated twice in chilled condition and centrifuged to remove tissue/cell debris. Protein concentration in the lysates was estimated using the Bradford method after TCA precipitation and 25–40 µg of protein was resolved on 8–10% SDS-PAGE, followed by electrophoretic transfer to PVDF membrane. The membrane was blocked with 5% bovine serum albumin (BSA) (Hi-Media, India) prepared in tris-buffered saline (pH 8.0) with 0.1% Tween-20 for one hour at room temperature followed by overnight incubation in the respective primary antibodies. The membrane was incubated with the respective secondary antibodies conjugated with HRP (Table A3) at room temperature for 1 h. Four washes of 1% PBST was given before and after secondary antibody incubation. The membrane was visualized with the clarity Western ECL substrate (Bio-Rad Laboratories, Hercules, CA, USA) in ChemiDoc™ Touch Imaging System. The immunoblot band intensities were quantified using Image lab^TM^ software (Bio-Rad Laboratories, Hercules, USA) and expressed as the molecule/β-actin ratio.

### 2.8. Statistical Analyses

The data generated were checked for normal distribution using column statistics. Most of the data were normally distributed and the difference between groups was calculated by one-way ANOVA followed by Tukey’s test for multiple-group analysis. The Kruskal–Wallis test was carried out for the data which was not normally distributed and a Dunn’s post hoc test for the multiple group comparison. Results are expressed as the mean ± SEM and *p* < 0.05 was considered as significant. All the analyses were carried out in GraphPad Prism 5 software. 

## 3. Results

### 3.1. Animals and Experimental Strategy to Develop T2D Mouse Model

**Figure A2:**
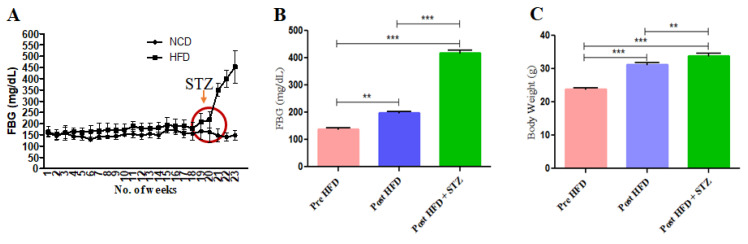
(**A**) Fasting blood glucose (FBG) levels in insulin-resistance mouse model. STZ administered at the 20th week showed FBG > 240 mg/dL. (**B**) FBG levels in experimental diabetic mice (pre- and post-HFD, and HFD + STZ treated mice). A significant difference in FBG levels was observed in the animals before and after HFD, and HFD + STZ treatment as compared to NCD mice (** *p* < 0.01, *** *p* < 0.001, respectively). (**C**) Body weight of experimental diabetic mice (pre- and post-HFD, and HFD + STZ treated). A significant difference in the body weight was observed in the HFD and HFD + STZ mice as compared to NCD mice (** *p* < 0.01, *** *p* < 0.001, respectively).

After 20 weeks of HFD treatment, animals became obese and insulin-resistant. After STZ administration, the FBG levels of these animals surpassed 240 mg/dL (Figure A2A). There was a significant increase in the FBG levels and BW post STZ stimulus (Figure A2B,C). 

### 3.2. Assessment of Metabolic and Biochemical Parameters

**Figure 1:**
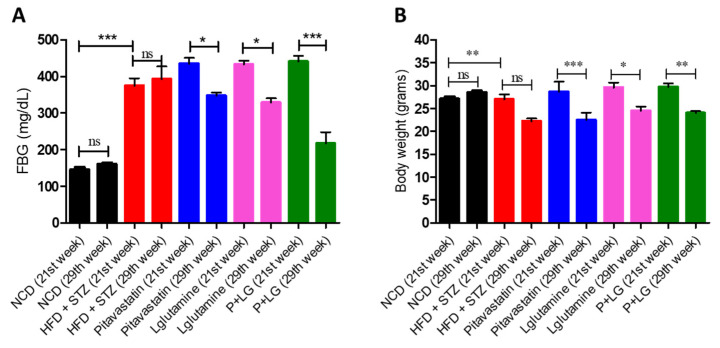
Assessment of metabolic and biochemical parameters. (**A**) Fasting blood glucose (FBG). (**B**) Body weight (BW). The 20 weeks group when compared to their respective 29 weeks groups (n = 5 per group). * *p* < 0.05, ** *p* < 0.01, *** *p* < 0.001.

There was significant rise in the FBG levels and body weight in the HFD + STZ group (*p* < 0.001 and *p* < 0.01) as compared to NCD. At the end of six weeks of drug treatment significant reduction in FBG levels of P (*p* < 0.05), LG (*p* < 0.05), and P + LG (*p* < 0.001) groups, (Figure 1A) and significant decrease in the body weight of P (*p* < 0.001), LG (*p* < 0.05), and P + LG (*p* < 0.01) groups as compared to the HFD + STZ group (Figure 1B) were observed.

#### 3.2.1. Intraperitoneal Glucose-Tolerance Test (IPGTT) and Intraperitoneal Insulin-Sensitivity Test (IPIST)

**Figure 2:**
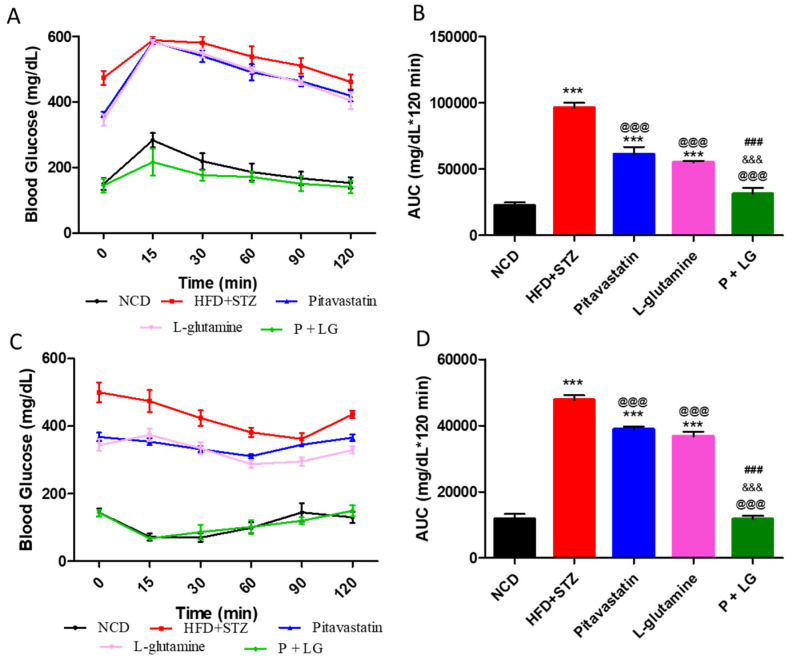
(**A**,**B**) Intraperitoneal glucose-tolerance test (IPGTT) and (**C**,**D**) intraperitoneal insulin-sensitivity test (IPIST) in the drug-treated animals. Data are reported as mean ± standard error of the mean for the test performed on the 29th week (n = 5 per group). (* signifies comparison against the NCD group, @ signifies comparison against the HFD + STZ group, ^&^ signifies comparison against the pitavastatin group, and ^#^ signifies comparison against the L-glutamine group, The number of symbols corresponds to *p* < 0.05, *p* < 0.01, *p* < 0.001).

The HFD + STZ group showed a sharp rise in FBG levels post glucose administration which took a longer time to resolve back to its basal level. Monotherapy-treated groups showed higher base-line glucose after four hours fasting. However, in spite of a sharp rise in the glucose levels of monotherapy groups the drop at 30 min and thereafter was faster, indicating their improved glucose tolerance as compared to the HFD + STZ group. The basal FBG levels in mice treated with P + LG were significantly lower and coincided with the NCD group as compared to the HFD + STZ group (Figure 2A). The area under the curve (AUC) of the 0–120 curve indicated improved glucose tolerance in the P + LG group as compared to the HFD + STZ group (Figure 2B). FBG levels in the P + LG group were significantly lower when compared to the P (*p* < 0.001) and LG (*p* < 0.001) groups after 60, 90, and 120 min of insulin administration (Figure 2C). AUC of the 0–120 curve in the P + LG group was significantly lower as compared to the HFD + STZ group after 60, 90, and 120 min of insulin administration (Figure 2D). 

#### 3.2.2. Lipid Profiling

**Figure 3:**
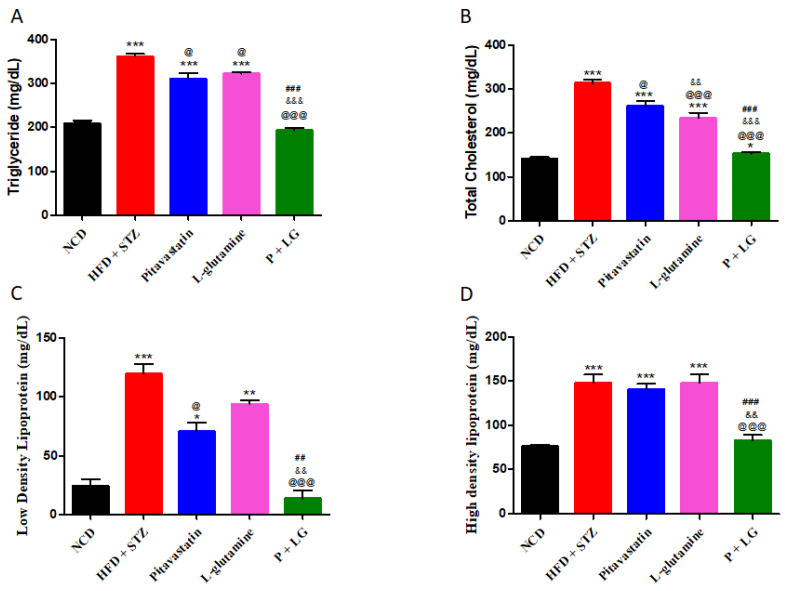
Plasma lipid profile: (**A**) triglyceride levels, (**B**) total cholesterol levels, (**C**) low-density lipoprotein levels, and (**D**) high-density-lipoprotein levels. Data are reported as mean ± standard error of the mean (n = 5 per group). (* signifies comparison against the NCD group, @ signifies comparison against the HFD + STZ group, ^&^ signifies comparison against the pitavastatin group, and ^#^ signifies comparison against the L-glutamine group, The number of symbols corresponds to *p* < 0.05, *p* < 0.01, *p* < 0.001).

Triglycerides (TG), total cholesterol (TC), low density lipoprotein (LDL), and high-density lipoprotein (HDL) levels were significantly increased in the HFD + STZ group (*p* < 0.001, *p* < 0.001, *p* < 0.001, and *p* < 0.05, respectively), as compared to the NCD group. The increased HDL is indicative of increased reverse cholesterol transport through the HDL pathway, as an adaptation to the metabolic load of a high-fat diet (Hayek et al., 1993). Upon six weeks of drug treatment the TG, TC, and LDL levels were significantly reduced in the P + LG group (*p* < 0.001, *p* < 0.001, and *p* < 0.001, respectively), as compared to HFD + STZ group (Figure 3A–C). Further, HDL levels were significantly increased in the P and LG groups but were significantly decreased (*p* < 0.001) in the P + LG group as compared to HFD + STZ group and were at par with the HDL levels in the NCD group (Figure 3D).

#### 3.2.3. Plasma Insulin and Adiponectin Levels

**Figure 4:**
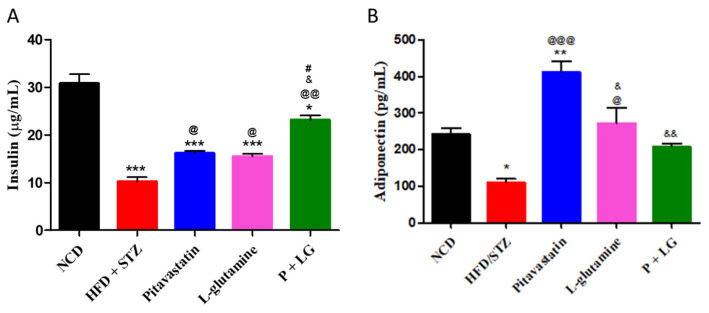
Plasma insulin and adiponectin levels: (**A**) insulin levels and (**B**) adiponectin levels. Data are reported as mean ± standard error of the mean (n = 5 per group). (* signifies comparison against the NCD group, @ signifies comparison against the HFD + STZ group, ^&^ signifies comparison against the pitavastatin group, and ^#^ signifies comparison against the L-glutamine group, The number of symbols corresponds to *p* < 0.05, *p* < 0.01, *p* < 0.001).

A significant decrease in insulin and adiponectin levels was observed in the HFD + STZ group (*p* < 0.001 and *p* < 0.05) as compared to the NCD group. After six weeks of drug treatment significant increase in the insulin levels was observed in P (*p* < 0.05), LG (*p* < 0.05) and P + LG (*p* < 0.01) groups (Figure 4A). Further, the adiponectin levels were significantly increased in P (*p* < 0.001) and LG (*p* < 0.05) groups while a normo-adiponectinemia was seen in the P + LG group (Figure 4B).

### 3.3. Gene Expression of GLUT2 and Glucoregulatory Enzymes and Their Activities in the Liver

**Figure 5:**
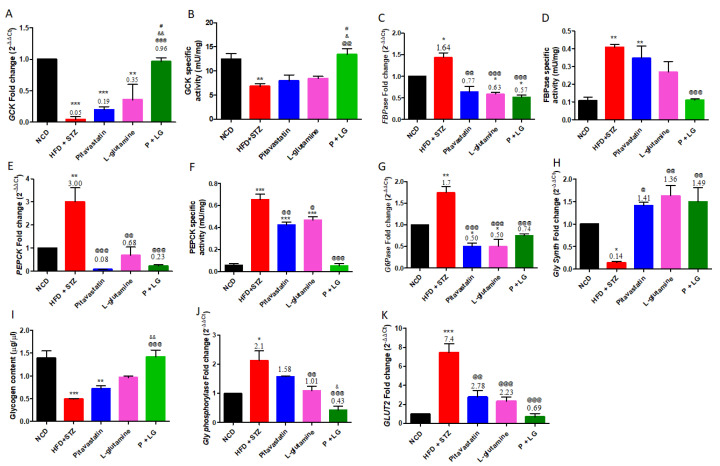
Glucoregulatory enzymes gene expression and activities in the liver: Glucokinase (GCK for glycolysis) (**A**) expression and (**B**) enzyme activity, fructose-1,6-bisphosphatase (FBPase for gluconeogenesis) (**C**) expression and (**D**) FBPase activity, phosphoenolpyruvate carboxykinase (PEPCK for gluconeogenesis) (**E**) expression and (**F**) PEPCK enzyme activity, (**G**) glucose 6 phosphatase (G6Pase for gluconeogenesis) expression, (**H**) glycogen synthase (glycogenesis) expression; (**I**) glycogen content; (**J**) glycogen phosphorylase (glycogenolysis) expression, and (**K**) GLUT2 gene expression. Data are reported as mean ± standard error of the mean (n = 4–5 per group). (* signifies comparison against the NCD group, @ signifies comparison against the HFD + STZ group, ^&^ signifies comparison against the pitavastatin group, and ^#^ signifies comparison against the L-glutamine group, The number of symbols corresponds to *p* < 0.05, *p* < 0.01, *p* < 0.001).

The gene expression, and activity of key enzymes involved in the glucoregulatory metabolic pathways were assessed in the liver. The HFD + STZ group showed significant decrease in GCK (glycolysis) expression (*p* < 0.001) and activity (*p* < 0.01) as compared to the NCD group (Figure 5A,B). There was also significant increase in the FBPase and PEPCK (gluconeogenesis) expression (*p* < 0.05, *p* < 0.01) and activity (*p* < 0.01, *p* < 0.001) (Figure 5C–F). In addition, the G6pase (gluconeogenesis) expression was increased in the HFD + STZ group (*p* < 0.01) while reduced glycogen synthase expression (*p* < 0.05), glycogen content (*p* < 0.01), and increased glycogen phosphorylase (glycogenolysis) and GLUT2 expression (*p* < 0.05 and *p* < 0.001) were observed in the HFD + STZ group. 

The P + LG group showed a significant increase in GCK expression (*p* < 0.01) and activity (*p* < 0.01) (Figure 5A,B) and increased expression of glycogen synthase (glycogenesis) (*p* < 0.01; Figure 5H), and increased glycogen content (*p* < 0.001; Figure 5I). Further, a significant decrease in the expression and activity of FBPase and PEPCK (*p* < 0.05, *p* < 0.001 and *p* < 0.001, *p* < 0.001 respectively; Figure 5C–F) along with a concomitant decrease in G6Pase expression (*p* < 0.01; Figure 5G) were observed in the P + LG group. Additionally, the glycogen phosphorylase expression was also reduced (*p* < 0.001; Figure 5J) in the combination-treated group with a concomitant decrease in GLUT2 receptor expression (*p* < 0.001; Figure 5K). 

### 3.4. Mitochondrial Biogenesis Marker Gene Expression Levels in Skeletal Muscle

**Figure 6:**
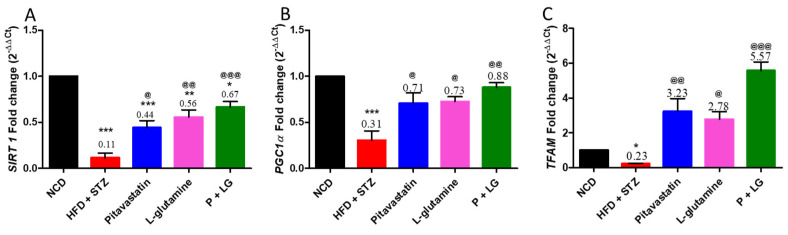
Mitochondrial biogenesis marker levels in skeletal muscle: (**A**) SIRT1, (**B**) PGC1α, and (**C**) TFAM. Data are reported as mean ± standard error of the mean (n = 5 per group). (* signifies comparison against the NCD group, @ signifies comparison against the HFD + STZ group, ^&^ signifies comparison against the pitavastatin group, and ^#^ signifies comparison against the L-glutamine group, The number of symbols corresponds to *p* < 0.05, *p* < 0.01, *p* < 0.001).

A significant decrease in the expression of SIRT1, PGC1α, and TFAM was observed in the HFD + STZ group as compared to the NCD group (*p* < 0.001, *p* < 0.001, and *p* < 0.05, respectively; Figure 6A–C). A significant increase in the expression of SIRT1 was observed in the monotherapies (*p* < 0.05 and *p* < 0.01) and the P + LG (*p* < 0.001) groups (Figure 6A) along with a significant increase in the expression of PGC1α and TFAM in the monotherapies (*p* < 0.05, *p* < 0.05, and *p* < 0.01, *p* < 0.05, respectively), and the P + LG (*p* < 0.01 and *p* < 0.001, respectively) groups (Figure 6B,C). 

### 3.5. Estimation of Oxygen Consumption Rate (OCR)

**Figure 7:**
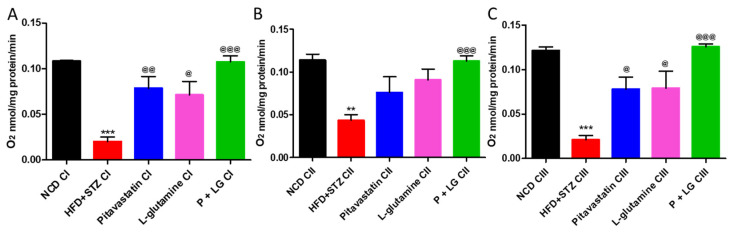
State3/state 4: (**A**) Complex I: OCR of state 3/state 4 in HFD + STZ group was reduced as compared to the NCD group. There was a rise in the OCR in the P + LG group. (**B**) Complex II: OCR of state 3/state 4 in HFD + STZ group was reduced as compared to the NCD group. There was a rise in the OCR in the P + LG group. (**C**) Complex III: OCR of state 3/state 4 in HFD + STZ group was reduced as compared to the NCD group. There was a rise in the OCR in the P + LG group. (* signifies comparison against the NCD group, @ signifies comparison against the HFD + STZ group, ^&^ signifies comparison against the pitavastatin group, and ^#^ signifies comparison against the L-glutamine group, The number of symbols corresponds to *p* < 0.05, *p* < 0.01, *p* < 0.001).

The OCR of state 3/state 4 in HFD + STZ group was significantly reduced as compared to the NCD group for the mitochondrial complexes I, II, and III (*p* < 0.001, *p* < 0.01, and *p* < 0.001, respectively; Figure 7A–C). A significant increase in the OCR of P, LG, and P + LG groups was observed in CI (*p* < 0.01, *p* < 0.05, and *p* < 0.001; Figure 7A) and CII (*p* < 0.05, *p* < 0.05, and *p* < 0.001; Figure 7B), respectively. However, the OCR of state 3/state 4 in complex III was significantly improved only in the P + LG group (*p* < 0.001, *p* < 0.001, and *p* < 0.001, respectively; Figure 7C) as compared to the HFD + STZ group. 

### 3.6. Western Blot Analysis

**Figure 8:**
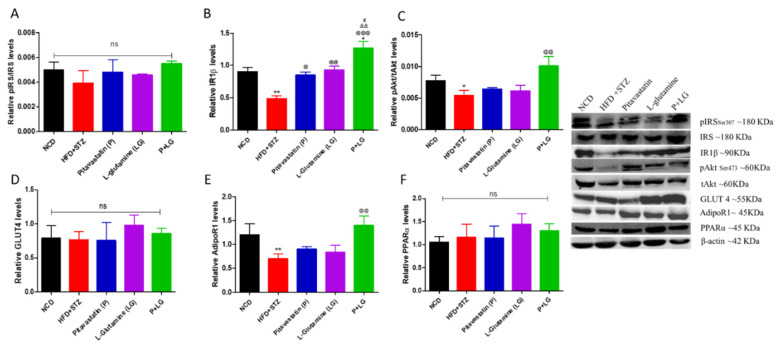
Insulin signaling pathway: (**A**) pIRS/IRS ratio, (**B**) insulin receptor1β, (**C**) pAkt/tAkt ratio, (**D**) GLUT4, (**E**) AdipoR1, and (**F**) PPARα. (* signifies comparison against the NCD group, @ signifies comparison against the HFD + STZ group, ^&^ signifies comparison against the pitavastatin group, and ^#^ signifies comparison against the L-glutamine group, The number of symbols corresponds to *p* < 0.05, *p* < 0.01, *p* < 0.001) (n = 3/group).

The levels of key insulin-signaling-pathway molecules were estimated by Western blot. There was significant decrease in the IR1β, pAkt/tAkt, and AdipoR1 expression levels in the HFD + STZ group (*p* < 0.01, *p* < 0.05, and *p* < 0.01, respectively; Figure 8B,C,E). The protein levels were restored in the combination-treated group (*p* < 0.001, *p* < 0.01, and *p* < 0.01, respectively; Figure 8B,C,E). Further, there was a significant increase in the IR1β levels in the P and LG groups as compared to the HFD + STZ group (*p* < 0.05 and *p* < 0.01, respectively; Figure 8B). However, no significant difference was observed in the pIRS/IRS ratio, GLUT 4, or PPARα levels (ns = non-significant; Figure 8A,D,F).

### 3.7. Regeneration and Apoptosis Analysis in Pancreatic β-Cells

**Figure 9:**
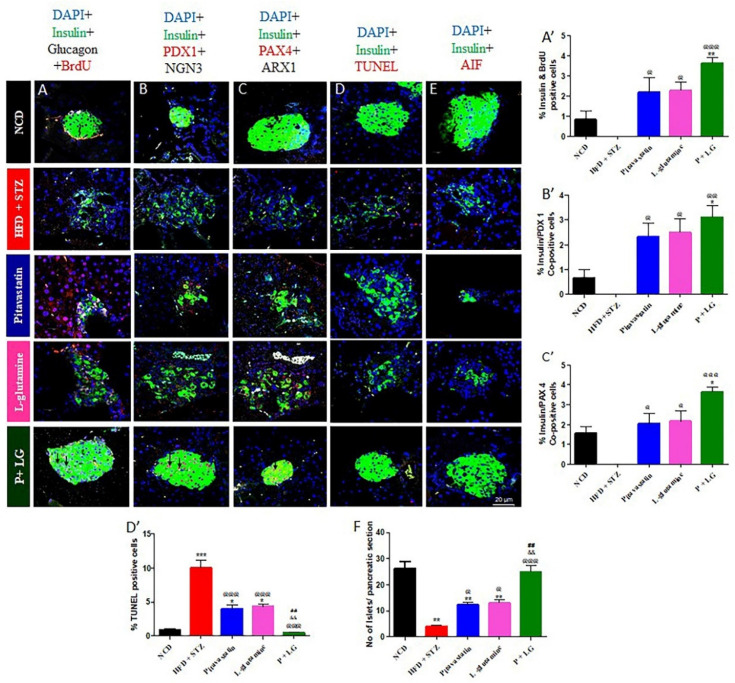
Immuno-histochemistry of pancreatic islets of Langerhans at 60×: (**A**,**A′**) proliferation, (**B**,**B′**) neogenesis (insulin/Pdx1/Ngn3), (**C**,**C′**) transdifferentiation (insulin/Pax4/Arx1), (**D**,**D′**) TUNEL, (**E**) AIF, and (**F**) number of islets/pancreatic section. (* signifies comparison against the NCD group, @ signifies comparison against the HFD + STZ group, ^&^ signifies comparison against the pitavastatin group, and ^#^ signifies comparison against the L-glutamine group, The number of symbols corresponds to *p* < 0.05, *p* < 0.01, *p* < 0.001). (n = 3/group, scale= 20µm, magnification = 60×).

A significant destruction of insulin-positive β-cells was seen in the HFD + STZ group with a significant increase in the number of apoptotic β-cells (*p* < 0.001; Figure 9A–E) and reduced number of islets/pancreatic section (*p* < 0.01; Figure 9F) as compared to the NCD group. Upon treatment there was a significant rise in the insulin/BrdU co-positive cells in P, LG, and P + LG groups as compared to the HFD + STZ group (*p* < 0.05, *p* < 0.05, and *p* < 0.001, respectively; Figure 9A,A’). Further, markers for neogenesis (Pdx1 and Ngn3), and transdifferentiation (Pax4 and Arx) were co-stained with insulin and it was observed that only Pdx1 and Pax4 were co-stained with insulin (*p* < 0.05, *p* < 0.05, and *p* < 0.01, and *p* < 0.05, *p* < 0.51, and *p* < 0.001, respectively; Figure 9B,B’ and C,C’, respectively) upon mono- and combination treatment. No Ngn3- or Arx-positive cells were found. There was a significant increase in the insulin/TUNEL positive cells in the HFD + STZ group (*p* < 0.001) which was reversed upon the treatment with mono- and combination therapies (*p* < 0.001, *p* < 0.001, and *p* < 0.001, respectively; Figure 9D,D’). Further, a significant decrease in the number of islets (image not shown) was observed in the HFD + STZ group (*p* < 0.01; Figure 9F). However, no AIF translocation was observed in the HFD + STZ group (Figure 9E). Overall, there was a significant increase in the number of islets/pancreatic section in the combination-treated groups as compared to HFD + STZ group (*p* < 0.001; Figure 9F).

## 4. Discussion

**Figure 10:**
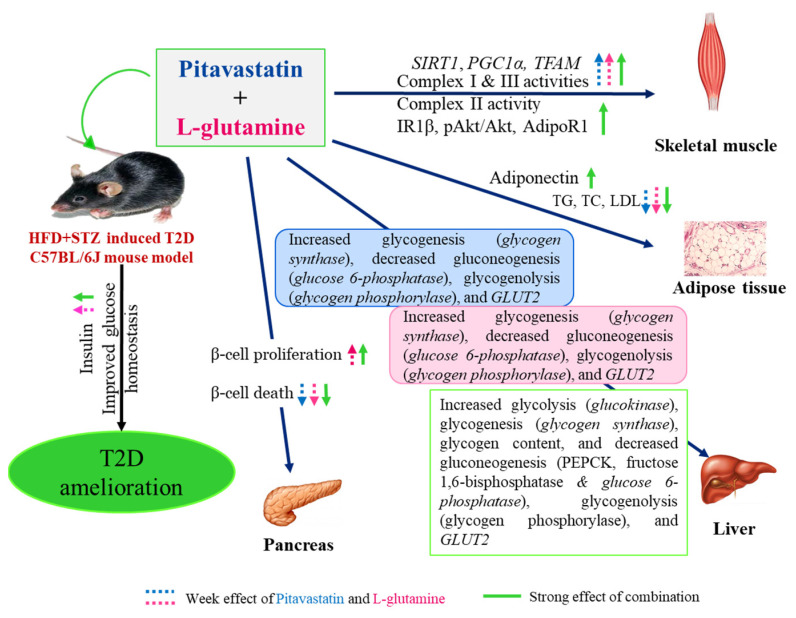
Effect of pitavastatin, L-glutamine, and the combination therapy on amelioration of T2D manifestations: Pitavastatin and L-glutamine induced normolipidemia and reduced the transcript levels of glucose 6-phosphatase, glycogen phosphorylase, and GLUT2 in the liver whilst increasing SIRT1, PGC1α and TFAM, and mitochondrial ETC complexes I and III activities in skeletal muscle. Both pitavastatin and L-glutamine also reduced β-cell death individually and L-glutamine also induced β-cell proliferation. In combination they additionally increased mitochondrial ETC complex III state 3/state 4 OCR in skeletal muscle, and glucokinase transcript levels with a concomitant decrease in PEPCK and fructose 1,6-bisphophatase activities in the liver. The combination therapy also induced normoadiponectinemia, increased pAkt/Akt, increased IR1β and AdipoR1 protein levels in skeletal muscle, and increased plasma insulin levels. Thus, the drugs together could bring about normolipidemia, reverse mitochondrial dysfunction, decrease gluconeogenesis, glycogenolysis, glycogen synthesis, and GLUT2 expression. The combination therapy could also reduce β-cell death, enhance glucose tolerance and insulin sensitivity thus being able to ameliorate T2D in HFD + STZ-induced mouse model.

Our results demonstrate that pitavastatin and L-glutamine in combination could ameliorate T2D manifestations by (1) improving glucose tolerance and insulin sensitivity, (2) normalizing the insulin and adiponectin levels, (3) inhibiting gluconeogenesis, reducing hepatic GLUT2 and enhancing glycolysis, and glycogen synthesis, (4) increasing the IR1β, pAkt, and AdipoR1 expression levels in skeletal muscle, (5) increasing mitochondrial biogenesis and ETC activities, and (6) increasing β-cell regeneration by proliferation (Figure 10).

Previous studies have reported the ability of statins in enhancing glucose tolerance and increasing anti-inflammatory adipokines by lowering LDLs and c-reactive proteins [20,21,22]. In vitro studies suggest that the off-target effect of pitavastatin on adiponectin may be related to the prevention of adipocyte hypertrophy and adipokine dysregulation [23]. Further, L-glutamine treatment has been reported to enhance glucose homeostasis, increase adiponectin levels, and reduce TG, TC, and LDL levels while increasing insulin secretion [24,25,26,27]. Thus, we hypothesized that the dramatic rise in the adiponectin levels of monotherapies to be a compensatory response for not being able to activate the adiponectin signaling pathway in our study. Going forward, we found that the protein expression of AdipoR1 was significantly increased only in the combination group. Although we did not see a significant change in the levels of PPARα, a downstream transcription factor activated upon adiponectin binding to AdipoR1, it can be suggested that the combination treatment enhanced insulin levels, glucose tolerance, insulin sensitivity, and FBG levels, and improved lipid profile as a consequence of the increased adiponectin signaling pathway. With increased adiponectin, hepatic glucose output is suppressed, lowering systemic glucose, enhancing the hepatic insulin sensitivity, and inhibiting the expression and activity of key enzymes involved in gluconeogenesis [28,29,30,31]. Thus, contrary to reports stating statins cause new-onset diabetes, we propose that statins do not lead to glucose overproduction in an already hyperlipidemic state of T2D when administered at low concentrations. We also report a significantly enhanced insulin-signaling pathway leading to glucose uptake in the mono- and combination therapy groups. This is the first report wherein the effect of pitavastatin on the insulin signaling pathway was studied in vivo. Although we did not find an increase in total GLUT4, the increased insulin sensitivity and glucose tolerance suggests an ultimate rise in the membrane translocated GLUT4.

Glucolipotoxicity is known to induce oxidative stress and mitochondrial dysfunction. Further, pitavastatin could increase the transcript levels of PGC1α, TFAM, and SIRT1, thus alleviating oxidative stress and enhancing the electron transport chain (ETC) complexes’ I and III activities as reinforced by previous studies [32]. For the first time, our results suggest that L-glutamine being an antioxidant, maintains mitochondrial integrity by inducing mitochondrial biogenesis, and increasing complex I and III activities. Finally, similar to our IHC results, Zhao and Zhao found that β-cells treated with low dose of pitavastatin were in the S and G2/M phase as compared to other statin groups in vitro [9]. Moullé et al. found that L-glutamine controlled the biosynthesis of IGF2, an autocrine regulator of β-cell mass and function, and activated Akt phosphorylation in β-cells leading to its proliferation [33]. L-glutamine has also been reported to be able to upregulate Pdx1 [34]. Pdx1 in concert with Ngn3 and Mafa is an important marker of islet neogenesis (differentiation of acinar cells to β-cells), while Pax4 is a marker of transdifferentiation (α- to β-cell conversion) [35,36] but we did not find Ngn3 and insulin co-positive cells. Hence, our data indicate significant increase in β-cell numbers via the proliferative pathway alone. Although there was only a marginally significant increase in the number of islets in monotherapies, they showed an increase in Pdx1 and Pax4 insulin co-positive cells demonstrating their individual β-cell regenerative properties. Pax4 was also reported to promote differentiation of Pdx1-positive mesenchymal stem cells to β-cell fate [37]. Future studies in this direction are warranted. 

We hypothesize that the individual adiponectin-enhancing properties of pitavastatin and L-glutamine, coupled with their combinatorial AdipoR1 expression-enhancing property resulted in the significant correction of T2D manifestations. Although several preclinical and clinical studies have been carried out to explore the individual therapeutic potential of pitavastatin and L-glutamine to ameliorate T2D, this is the first study to explore the combination effect of the two drugs on various diabetes manifestations. 

Future studies are needed to understand the mechanism of adiponectin potentiating action, increasing AdipoR1 expression and β-cell proliferation of the combination therapy.

## 5. Conclusions

Pitavastatin and L-glutamine in combination could ameliorate T2D manifestations acting together at hepatic, skeletal muscle, and pancreatic fronts.

## Data Availability

Data is available on request to the corresponding author.

## Appendix A

**Table A1:** Primers for the transcript analysis.

Gene Primer	Sequence (5′-3′)	Annealing Temperature	Amplicon Size (bp)	Tissue
Glucokinase	FP: AGGAGGCCAGTGTAAAGATGT RP: TCCCAGGTCTAAGGAGAGAAA	56 °C	90 bp	Liver
PEPCK	FP: CTGCATAACGGTCTGGACTTC RP: CAGCAACTGCCCGTACTCC	65 °C	151 bp	
G6Pase	FP: CTGTTTGGACAACGCCCGTAT RP: AGGTGACAGGGAACTGCTTTA	56 °C	91 bp	
Glut2	FP: CTTGGAAGGATCAAAGCAATG RP: CAGTCCTGAAATTAGCCCAC	60 °C	150 bp	
Glycogen synthase	FP: ACCAAGGCCAAAACGACAG RP: GGGCTCACATTGTTCTACTTG	61 °C	102 bp	
Glycogen phosphorylase	FP: GAGAAGCGACGGCAGATCA RP: CTTGACCAGAGTGAAGTGCA	65 °C	102 bp	
Fructose 1,6, bisphosphatase	FP: GCATCGCACAGCTCTATGGT RP: CTCAGGTTCGATTATGATGGC	63 °C	120 bp	
SIRT-1	FP: GATGAAGTTGACCTCCTCA RP: GGGTATAGAACTTGGAATTAG	64 °C	86 bp	Skeletal Muscle
PGC-1α	FP: AGCCGTGACCACTGACAACGA RP: GTAGCTGAGCTGAGTGTTGGC	69 °C	129 bp	
TFAM	FP: CTGAGGAAAAGCAGGCATA RP: ATGTCTCCGGATCGTTTCAC	69 °C	142 bp	
GAPDH	FP: AGGTCGGTGTGAACGGATTTG RP: TGTAGACCATGTAGTTGAGGT	56 °C	123 bp	Liver, Skeletal muscle

FP: forward primer; RP: reverse primer; bp: base pair.

**Table A2:** Details of the antibodies used for the IHC studies.

Primary Antibody	Secondary Antibody	Excitation (nm)	Emission (nm)
Anti-insulin (1:200, guinea pig) (DAKO Agilent, USA)	Anti-guinea pig Alexa 568 (1:500, Donkey) (Jackson ImmunoResearch Laboratories, Inc. USA)	493	519
Anti-NGN3 (1:50, rabbit) (Thermo Fisher Scientific, USA)	Anti-rabbit Alexa 647 (1:500, Donkey) (Jackson ImmunoResearch Laboratories, Inc. USA)	651	667
Anti-ARX (1:500, rabbit) (Sigma-Aldrich, Germany)			
Anti-AIF (1:400, rabbit) (Cayman chemicals, USA)			
Anti-glucagon (1:200, rabbit) (Cell Signaling Technology, USA)			
Anti-PDX-1 (1:1000, goat) (Abcam, USA)	Anti-goat Rhodamine Red X (1:200, Donkey) (Jackson ImmunoResearch Laboratories, Inc. USA)	570	590
Anti-PAX-4 (1:500, Goat) (Sigma-Aldrich, Germany)			
Anti-BrdU (1:100, rat) (Abcam, USA)	Anti-rat Rhodamine Red (1:200, Donkey) (Jackson ImmunoResearch Laboratories, Inc. USA)	570	590

PDX1: pancreatic and duodenal homeobox 1; ARX1: aristaless-related homeobox-encoding gene; PAX4: paired box 4; NGN3: neurogenin 3; BrdU: 5-bromo-2’-deoxyuridine.

**Table A3:** Details of the antibodies used for the immunoblot studies.

Primary Antibody	Secondary Antibody
Anti-β-actin (1:10,000, mouse) (ABclonal Technology, USA)	Anti-mouse IgG-HRP (1:10,000, goat) (Santa Cruz Biotechnology)
Anti-insulin receptor β (1:1000, rabbit) (Cell Signaling Technology, USA)	Anti-rabbit immunoglobulin G (IgG)-horse radish peroxidase (HRP) (1:5000, goat) (Santa Cruz Biotechnology)
Anti-insulin receptor substrate-1 (1:1000, rabbit) (ABclonal Technology, USA)	
Anti-phosphorylated insulin receptor substrate -1 Ser307 (1:1000, rabbit) (Cell Signaling Technology, USA)	
Anti- Akt-1 (Protein kinase B) (1:1000, rabbit) (ABclonal Technology, USA)	
Anti-phosphorylated Akt-1(Protein kinase B) S473 (1:1000, rabbit) (ABclonal Technology, USA)	
Anti-Glut-4 (Glucose transporter type 4) (1:1000, rabbit) (MERCK Millipore)	
Anti-AdipoR1 (Adiponectin receptor 1) (1:1000, rabbit) (Bioss Antibodies, USA)	
Anti-PPARa (Peroxisome proliferator activated receptor alpha) (1:1000, rabbit) (Bioss Antibodies, USA)	

**Table A4:** Nutritional composition of normal chow-diet and high-fat diet.

Nutritional Composition	Normal-Chow Diet (NCD) (%)	High-Fat Diet (HFD) (%)
Protein	22.5	18.15
Fat	5	45.55
Carbohydrate	55	24.19
Fiber	7	3.1
Ash	6.5	3.8
Moisture	4	3.5
